# Assessment of wear characteristics, longevity and stiffness of Essix-type retainers

**DOI:** 10.1007/s00784-024-05503-x

**Published:** 2024-03-02

**Authors:** Lina Alfadil, Mangala Patel, Nikolaos Pandis, Padhraig S. Fleming

**Affiliations:** 1 Queen Mary University of London, London, E1 4NS UK; 2 Centre Lead for Oral Bioengineering, Queen Mary University of London, Mile End Road, London, E1 4NS UK; 3 Universität Bern, Bern, Switzerland; 4 Chair/Professor of Orthodontics, Division of Public and Child Dental at Trinity College Dublin, Dublin Dental University Hospital, Lincoln Place, Dublin 2, D02 F859 Ireland

**Keywords:** VFRs, Retainers, Essix, Orthodontic, Retention, Thermoplastic

## Abstract

**Objective:**

To compare four commercially available Essix-type retainers in terms of longevity, wear characteristics, stiffness and their range of rigidity.

**Materials and methods:**

An *in* v*itro* study was conducted at Queen Mary University of London. Four groups of thermoplastic materials were included: Duran (PETG), Essix C + (Polypropylene), Vivera and Zendura (Polyurethane). A working typodont was fabricated to evaluate surface wear characteristics using a wear machine with a customized jig. Retainers were measured for tensile test, and water absorption was measured at five different time points up to 6 months after initial immersion in two different physical states and two different solutions. Hydrolytic degradation was also evaluated using FTIR spectroscopy.

**Results:**

Essix C + was the most flexible retainer with Vivera the stiffest material. Zendura and Essix C + had the most surface wear (413 μm ± 80 and 652 μm ± 12, respectively) with absorption rates of up to 15 wt% in artificial saliva occurring with Zendura. Only Essix C + displayed signs of degradation following water absorption.

**Conclusions:**

All materials had characteristic levels of flexibility and were susceptible to water absorption. Duran 1.5 mm performed similarly to Vivera in relation to stiffness and wear properties. While Zendura and Vivera have similar chemical structures, they exhibited differences concerning wear resistance and water absorption. Further clinical research evaluating the clinical relevance of these laboratory findings is required.

**Clinical relevance:**

Characteristic patterns of wear and rigidity of four commercially available Essix-type retainers were observed. This information should help in the tailoring of retainer material on a case-by-case basis considering treatment-related factors and patient characteristics including parafunctional habits.

## Introduction

The use of removable retention following orthodontic treatment is commonplace in order to mitigate against relapse related to treatment allied to maturational changes. Essix-type retainers are clear thermoplastic removable retainers first introduced in 1971 [1]. They were refined and popularized by Sheridan in 1993 [2] and are increasingly popular among orthodontists being the removable retainer of choice in the USA, UK, Ireland and Australia [3–7]. Their widespread adoption relates primarily to acceptable aesthetics, low cost and ease of fabrication.

Essix-type retainers are made from thermoplastic polymers that can be divided into two types: amorphous and semi-crystalline. Polypropylene (PP) is the most common semi-crystalline material used for Essix-type retainers. Amorphous polymers include polyethylene co-polymer (PETG), and more recently polyurethane polymer (PU). When these materials are tested under high temperatures exceeding their glass-transition temperature (*T*_*g*_), the polymer chains relax, separate and become mobile, making the material highly viscoelastic, which permits moulding into the shape required. As the material cools below that temperature threshold, hardening occurs. During the fabrication process, the retainers are formed through either a vacuumed or pressured heating cycle using blanks varying in thickness from 0.4 to 2mm.

The longevity of Essix-type retainers is known to be limited with a reported failure rate of 10% over a 2-year period [8] and minor fractures as well as loss also commonplace contributing to a lifespan of as little as 6 months based on one prospective study [9]. Thermoplastic materials are exposed to temperature variation in the intra-oral environment. This makes them susceptible to hydrolytic degradation, a process that affects polymers that are more water-absorbent in high-temperature states. The process of degradation is influenced by hydrophobic/hydrophilic properties, level of crystallinity, molecular weight, glass transition temperature (*T*_*g*_) and manufacturing procedure. Hence, different types of Essix-type retainer materials demonstrate characteristic mechanical properties and are vary in their propensity to degradation, wear and fracture. In view of the relative flexibility of Essix-type materials, alternatives including the use of metal-reinforced Essix-type retainers and substitution of Essix-type retainers for more rigid Hawley-type retainers have been advocated in order to maintain significant transverse change, particularly following active transverse expansion [10].

Previous studies have compared water absorption, wear resistance and post-fabrication morphology associated with Essix-type retainers. However, the mechanical properties of novel amorphous and semi-crystalline retainers are unclear. Moreover, the effect of varying retainer thickness on stiffness is yet to be investigated.

## Aim and hypothesis

To compare in vitro four commercially available Essix-type retainers in terms of longevity, wear characteristics and stiffness. Our null hypothesis dictates no difference exists between the types of materials with respect to longevity based on susceptibility to wear and degradation.

## Materials and methods

### Study design

A controlled laboratory-based investigation was undertaken within the Dental Physical Sciences Unit, on the Mile End Campus at Queen Mary University of London.

### Sample selection

Four different materials were compared: Essix C + (Raintree Essix, Inc., LA, USA), Vivera (Align Technology Inc., CA, USA), Zendura (BayMaterials LLC, Fremont, CA, USA) and Duran (SCHEU-Dental GmbH, Iserlohn, Germany) in two different thicknesses (1 mm and 1.5 mm). Vivera and Zendura are both polyurethane materials (PU), while Duran is a polyethylene co-polymer (PETG), and Essix C + is composed of polypropylene (Table 1). Five retainers were used in each group with a total of 25 retainers tested in this study.

**Table 1:** Thermoplastic materials and dimensions used in the study

Product	Dimensions (thickness)	Manufacturer	Composition
Essix C +	125 mm × 125 mm × 1 mm	Dentsply Raintree Essix, LA, USA	Polypropylene
Vivera	125 mm × 125 mm × 1 mm	Align Technology Inc., CA, USA	Polyurethane
Zendura	125 mm × 125 mm × 1 mm	Bay Materials LLC, Fremont, CA, USA	Polyurethane
Duran 1	125 mm × 125 mm × 1 mm	SCHEU-Dental GmbH, Iserlohn, Germany	Polyethylene terephthalate glycol (PETG)
Duran 1.5	125 mm × 125 mm × 1.5 mm	SCHEU-Dental GmbH, Iserlohn, Germany	Polyethylene terephthalate glycol (PETG)

### Retainer fabrication

An intra-oral scanner (7 Series, Straumann Group, Switzerland) was used to scan a typodont model (aligned U-shaped arch form) creating a 3D printed model to aid with the fabrication of three of the Essix-type retainers. To fabricate the Vivera retainers, an iTero intra-oral scanner (Align Technology Inc., CA, USA) was used. By following manufacturer guidelines, the Essix-type retainers were pressure-formed on the 3D printed models using a universal pressure-thermoforming unit (Dreve-Drufomat- TE/-SQ, Dreve-Dentamid, Germany). The Vivera retainers were fabricated separately by Align Technology.

### Mechanical testing procedures

#### Wear test

The retainers were cut into 25 samples (30 mm × 20 mm) using a digital calibrator targeting the second premolar-first molar region standardized on each sample, to fit into the steel plates housed in the wear testing machine. The retainers were cut into 25 samples (30 mm × 20 mm from each retainer sample), to fit into the steel plates housed in the wear testing machine. The cut samples were then flattened by oven heating at a temperature below the *T*_*g*_ of the materials (80 °C for 30 s) before being pressed for 10 s under a load of 2 kg. The post-thermoforming thickness of the Essix-type retainers may vary depending on the tooth surface (i.e. with greater thickness on the occlusal surfaces of the molars and canine region versus the labial surfaces of the teeth). Allowance was made for this with the average thickness for each sample recorded. The pre-cut specimens were placed on rectangular steel plates attached to the base of the wear testing machine (Boston Gear, Braintree, MA). A custom-made attachment was fabricated and attached to the extending metal rods of the wear machine with a load of 470 g, which consisted of 10 mm steatite balls embedded in light-cured acrylic.

A full cycle was represented by the movement of the attachments in a horizontal motion by 40 mm to the left followed by 40 mm to the right and ending in the starting position. Two thousand cycles were performed per specimen, requiring approx. 14 h in total. Between testing of each specimen, the machine and samples were cleaned with distilled water and air.

A three-dimensional, non-contact optic profilometry scanner (Proscan 2000; Scantron, Taunton, UK) with a resolution of 0.01 to 4 μm was used. Samples were scanned in an unworn state initially in order to account for initial surface irregularities. Scans were repeated after the wear process to permit assessment of the wear characteristics of the materials. An average of two readings was taken using the same reference points for all samples with surface wear measured in micrometres (μm). Each scan required a minimum of 45 min.

#### Water uptake and hydrolytic degradation

**Fig. 1:**
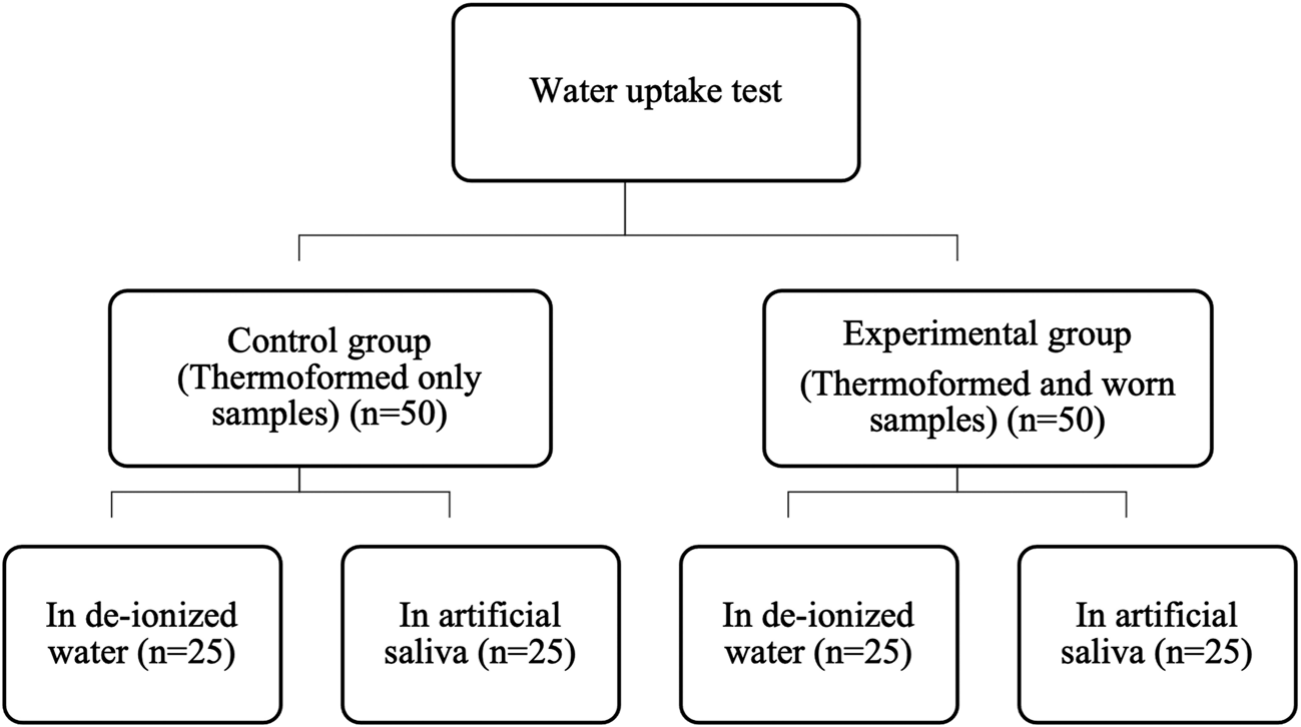
Sample distribution for hydrolysis test

For the hydrolytic uptake and degradation test, the worn samples were cut into even halves, producing 50 samples (15 mm × 10 mm) with uniform thickness (with the exception of Duran 1.5 mm). The thermoformed-only group also involved a digital calibrator to ensure similar location and dimension to those in the thermoformed and worn group. Thereafter, the samples were divided into two main groups—a control group, and an experimental group (Fig. 1). The control group consisted of retainers that had been thermoformed only, while the experimental group consisted of worn retainers evaluated after being subject to wear cycling. Each of the two groups was further divided as follows: Group 1 was immersed in 37 °C de-ionized water (pH level of 7.4), and Group 2 was immersed in artificial saliva at 37 °C. Proprietary artificial saliva was used (A.S. Saliva Orthana, CCMed, UK). Both groups were immersed and evaluated for water uptake at five intervals (T0: Baseline; T1: 12 h; T2: 24 h; T3: 720 h, i.e. 1 month; T4: 2160 h, i.e. 3 months; T5: 4320 h, i.e. 6 months).

Percentage water uptake was calculated using the equation:

$$\left(\mathrm{wt\%}\right)=\left[\left({w}_{i}-{w}_{o}\right)/{w}_{o}\right]\times 100$$

*w*_i_ and *w*_o_ are the weight of the specimen before and after uptake, respectively. For each reading, the specimen was blotted with filter paper to absorb water from the surface and then weighed using an electronic balance at room temperature (21 ± 1 °C). Reading accuracy was 0.0001 g, and variation in specimen weight was less than 0.1%.

For the degradation progress, Fourier transform infrared spectroscopy **(**FTIR) was used (PerkinElmer Frontier IR/FIR, PerkinElmer, UK**)** pre-testing (in the thermoformed state) and following cycling (T5) to assess the composition of the materials, degradation and changes in their chemical composition. Two samples were scanned twice to ensure homogeneity of the results with samples then dried for 1 week in a drying oven at 37 °C ± 1 °C, then re-scanned to confirm the results.

#### Tensile test

Thermoformed only retainer samples were cut into a dog-bone shape (70 mm × 7mm × 14 mm from each retainer sample, measured using a digital calibrator and cut with a surgical blade). The tensile strength test was performed using a universal mechanical testing instrument (Instron Co., Norwood, MA, USA) with a load cell of 3 kN at 37 °C. The distance between points was defined as 10 mm, and the crosshead speed was 0.2 mm/s in order to obtain stress–strain curves. Young’s elastic modulus (MPa) and tensile yield stress (MPa) were calculated from the obtained stress–strain curves.

### Statistical analysis

Descriptive analysis is presented for all experimental groups as mean values and standard deviations. To examine the effect of material on the yield and the Young’s module of elasticity linear regression was used and Scheffe’s method was applied for post hoc pairwise comparisons. For the effect of brand and time both on wear and water uptake adjusted for wear and solution a generalized estimating equation (GEE) model was used with robust standard errors. Linear regression analysis was used to assess the effect of brand on yield and Young’s modulus of elasticity. All analyses were conducted using Stata 17 (Stata Corp, TX, USA) and the R Software version 4.0.3 (R Foundation for Statistical Computing, Vienna, Austria). A *P* value of < 0.05 was considered statistically significant.

## Results

### Degree of surface wear on the materials

**Fig. 2:**
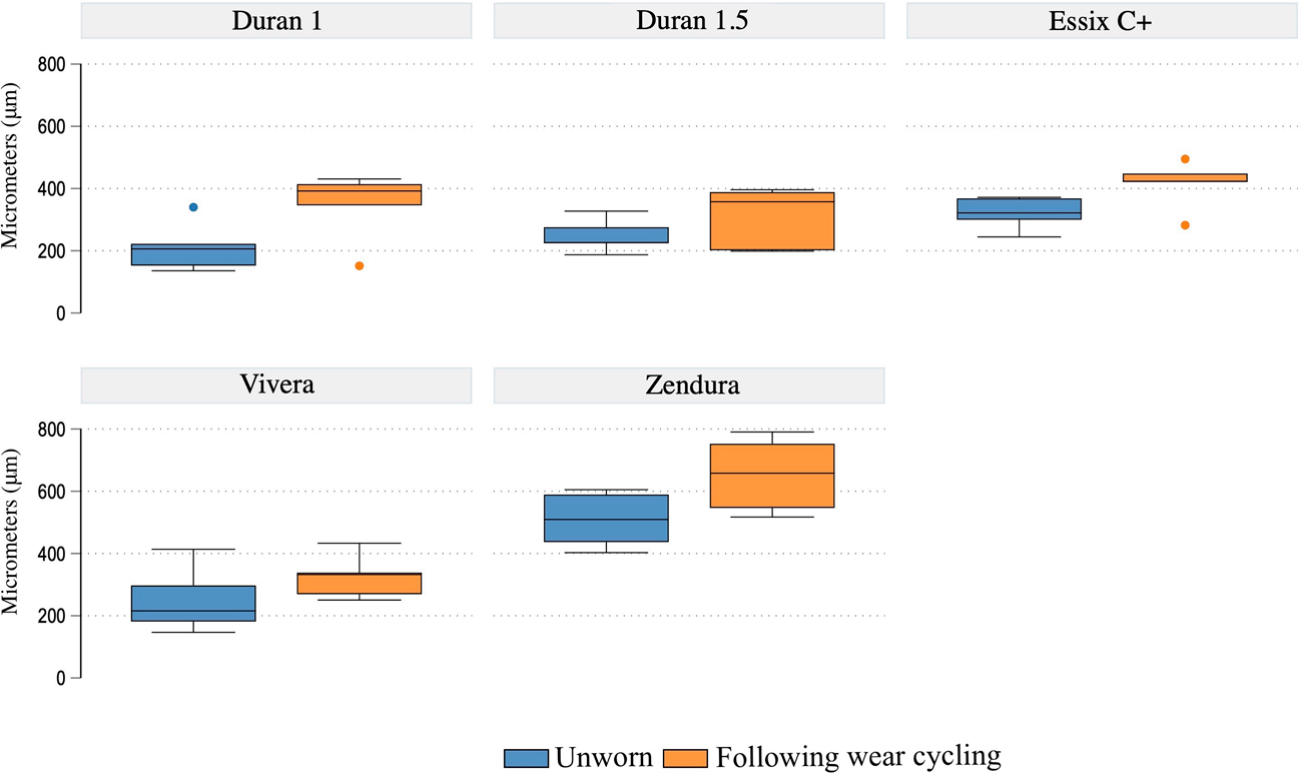
Boxplot of the surface irregularity in the unworn state and following wear cycling (in μm) for each retainer type

**Fig. 3:**
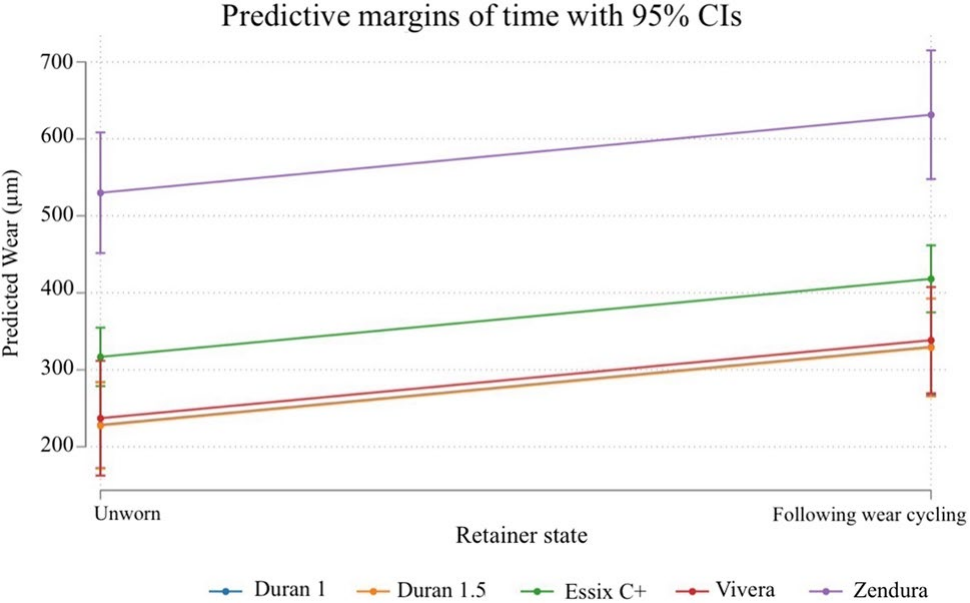
Predictive margins of time with 95% confidence intervals on the degree of surface wear (in μm) between the groups

Twenty-five thermoformed samples were scanned prior to and following wear cycling. Essix C + and Zendura exhibited the highest surface wear, averaging 413 μm ± 80 and 652 μm ± 12, respectively. Similar levels of wear were observed with Duran 1 mm and 1.5 mm (*P* = 0.9; Fig. 2). Vivera underwent less wear than Duran 1 (324 μm ± 71), while no significant difference was observed between Duran 1 mm, Duran 1.5 mm and Vivera in terms of wear rates (Table 2, Fig. 2). The results from the GEE model are shown in Table 3 with the Wald test for the main effects confirming that retainer material (*P* < 0.001) and time (*P* < 0.001) were significant wear predictors. A graphical display of the predicted effects is shown in Fig. 3.

**Table 2:** Descriptive data of surface irregularities in the unworn state and following wear cycling

Essix-type retainer	Mean (μm)	± SD	Median (μm; p50)	IQR
Duran 1				
Pre	211.19	80.40	205.80	69.80
Post	346.81	113.71	391.90	68.00
Duran 1.5				
Pre	248.12	54.34	226.20	51.30
Post	308.47	99.95	357.60	187.00
Essix C				
Pre	321.08	52.62	321.60	68.40
Post	413.88	79.45	423.35	26.90
Vivera				
Pre	250.86	106.72	215.65	115.75
Post	324.66	71.67	332.25	69.30
Zendura				
Pre	508.57	89.63	509.20	152.35
Post	652.89	121.06	657.95	205.95

**Table 3:** GEE analysis assessing the effect of material on surface wear adjusted for time

Surface wear	Coefficient	*P* value	95% confidence interval	
Duran 1.5 mm (base comparison)	Reference			
Duran 1 mm	0.70	0.98	− 79.76	81.17
Essix C +	89.19	0.01	21.24	157.13
Vivera	9.46	0.84	− 80.56	99.49
Zendura	302.44	0.00	204.92	399.95
Pre-wear (base comparison)	Reference			
Post-wear	101.38	0.00	65.58	137.18

### Water absorption and degradation properties of the materials

**Fig. 4:**
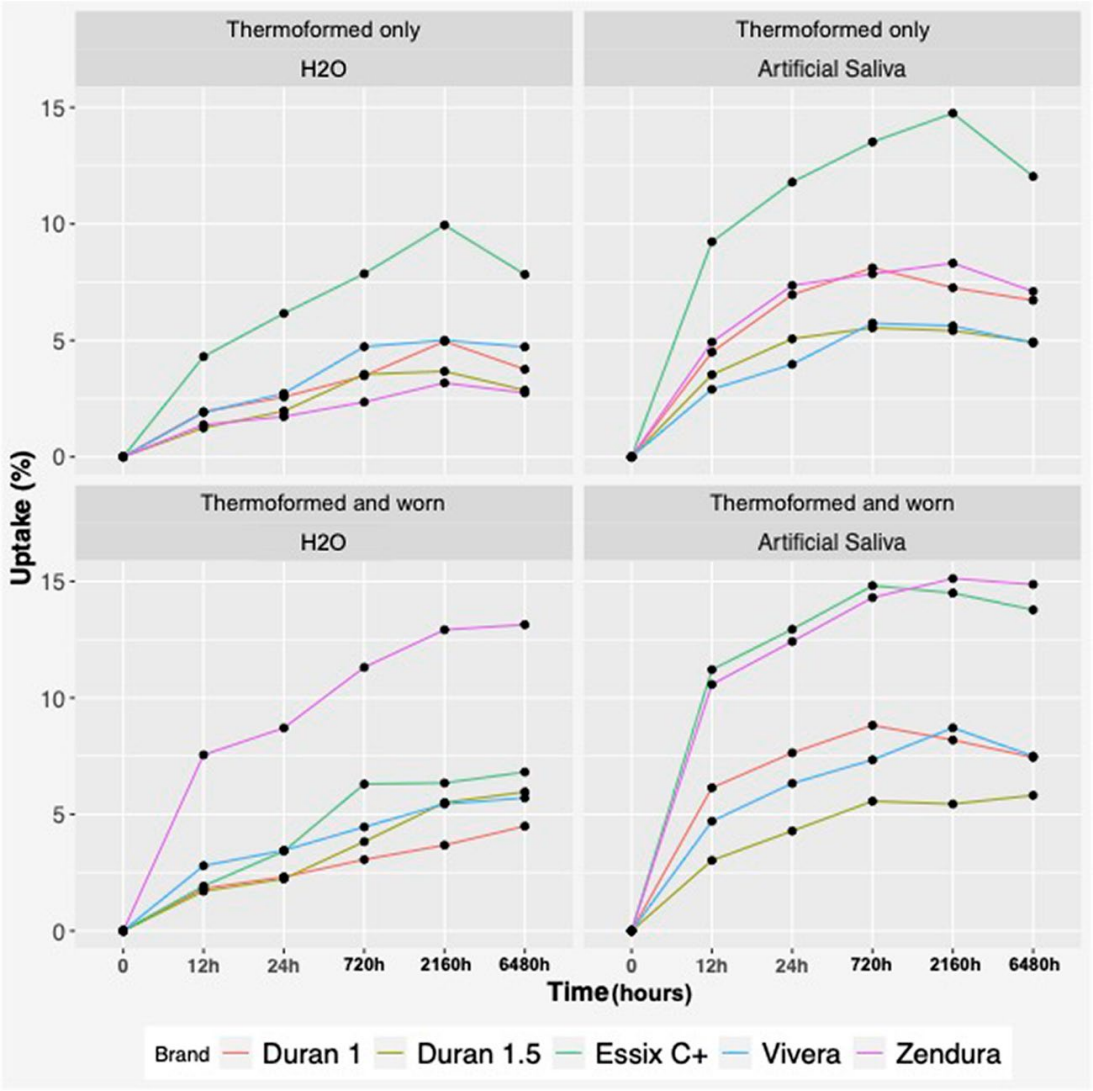
Graph demonstrating percentage water absorption in different groups

The amount of water absorbed is presented in Table 4 and 5 and shown graphically in Fig. 4. All samples experienced water uptake and reached a plateau (equilibrium) during the experiment at the 3-month time-point (T4).

**Table 4:** Water absorption (wt%) for the worn retainer group in grammes (experimental) in de-ionized water (H_2_O) and artificial saliva (AS)

Material	12 h		24 h		1 month		3 months		6 months	
	Mean wt%	± S.D	Mean wt%	± S.D	Mean wt%	± S.D	Mean wt%	± S.D	Mean wt%	± S.D
Essix C + H_2_O	0.19	0.01	0.20	0.01	0.20	0.01	0.20	0.01	0.20	0.01
AS	0.20	0.01	0.21	0.02	0.21	0.02	0.21	0.01	0.20	0.02
Vivera H_2_O	0.23	0.01	0.23	0.01	0.24	0.01	0.24	0.02	0.24	0.02
AS	0.23	0.01	0.24	0.01	0.24	0.01	0.24	0.01	0.24	0.01
Duran 1 H_2_O	0.25	0.02	0.26	0.02	0.26	0.02	0.26	0.02	0.26	0.01
AS	0.25	0.01	0.25	0.02	0.26	0.02	0.25	0.01	0.25	0.01
Duran 1.5 H_2_O	0.39	0.02	0.39	0.02	0.39	0.02	0.40	0.02	0.39	0.02
AS	0.38	0.04	0.39	0.03	0.39	0.03	0.39	0.03	0.38	0.03
Zendura H_2_O	0.26	0.01	0.26	0.01	0.26	0.01	0.26	0.01	0.26	0.01
AS	0.26	0.03	0.26	0.03	0.26	0.03	0.26	0.03	0.26	0.03

**Table 5:** Adjust GEE model for water uptake based on retainer type and time

Covariate	Coef	95% conf. interval	*P* value
Time			
0 h* (base comparison)	Reference		
12 h	0.010	(0.009 to 0.011)	< 0.001
24 h	0.014	(0.012 to 0.015)	< 0.001
1 m	0.017	(0.015 to 0.019)	< 0.001
3 m	0.018	(0.017 to 0.020)	< 0.001
6 m	0.017	(0.015 to 0.019)	< 0.001
Brand			
Duran 1* (base comparison)	Reference		
Duran 1.5	0.114	(0.103 to 0.125)	< 0.001
Essix C +	− 0.061	(− 0.065 to − 0.056)	< 0.001
Vivera	− 0.011	(− 0.014 to − 0.008)	< 0.001
Zendura	0.022	(0.020 to 0.024)	< 0.001
State			
Thermoformed* (base comparison)	Reference		
Worn	− 0.002	(− 0.006 to 0.002)	0.265
Solution			
Water* (base comparison)	Reference		
Artificial saliva	− 0.001	(− 0.003 to 0.001)	0.394

Overall, the worn (experimental) group absorbed more water compared to the thermoformed (control) group and samples immersed in artificial saliva absorbed more water in comparison to those immersed in de-ionized water (Table 4, Fig. 4). However, no significant difference in the absorption was noted based on wear cycling (*P* = 0.26). Essix C + group absorbed an average of 6 wt% in de-ionized water and up to 15 wt% in artificial saliva, for both the control (thermoformed only) and experimental (thermoformed and worn) groups (*P* < 0.01).

Zendura in the control group absorbed ~ 3 wt% de-ionized water, and more than double the amount was absorbed in artificial saliva (~ 8 wt%). A similar absorption pattern was seen with Zendura in the experimental group, with an increase to 13 wt% in de-ionized water and 15 wt% in artificial saliva. Vivera and Duran 1 mm performed in a similar manner throughout the different groups and solutions, averaging ~ 8% for maximum absorption. Meanwhile, the Duran 1.5 mm group had the lowest absorption values in all groups and solutions, reaching a peak of 6% in the worn state. The results of the adjusted GEE model are shown in Table 5. The overall Wald tests after fitting the GEE model showed that time and brand were significant predictors for water uptake (< 0.001).

With the exception of Essix C + (polypropylene), there was no difference in the FTIR spectra of the samples when compared between post-thermoforming, 6 months immersion, and 1 week of drying. Both Zendura and Vivera (polyurethane) displayed similar FTIR spectra confirming the polyurethane structure with the characteristic carbonyl absorption band of the ester bond located at 1750 cm^−1^ and a shoulder at 1656 cm^−1^ indicating a stretching vibration of carbonyl (C = O) group. The absorbance at 3305 cm^–1^ represents the stretching of the NH bond which is typically noted in urethane and urea groups. These bonds remained consistent throughout all timelines. The spectra for Duran (polyethylene terephthalate glycol) showed the characteristic bands of C–H stretching at 2906 cm^−1^ and 2866 cm^−1^, C = O at 1711 cm^−1^, and two peaks at 1410 cm^−1^ and 1240 cm^−1^ ascribed to –CH_2_– and C(O)–O stretching of ester groups, respectively.

**Fig. 5:**
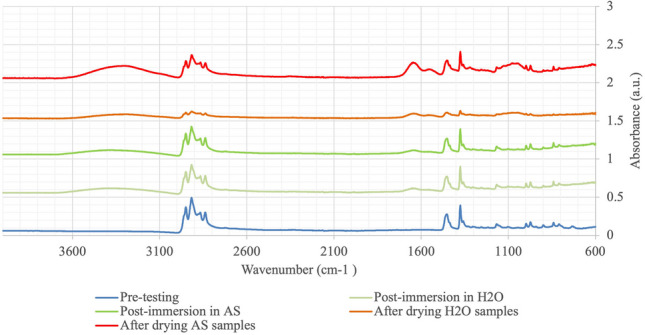
FTIR scan of Essix C + group at different time points

The FTIR spectrum of Essix C + displayed a shoulder at 2910 cm^−1^, asymmetric and symmetric in-plane C–H (–CH_3_) bond at 1446 cm^−1^, and the shoulder at 1372 cm^−1^ confirms that it is polypropylene. The peak at 1376 cm^−1^ is assigned to the –CH_3_ group. Additional absorption bands were found as broad O–H group stretch at 3300 cm^−1^ and 1611 cm^−1^, which can be attributed to stretching vibration of carbonyl (C = O) group that was noted following testing and drying (Fig. 5).

### Stiffness of the materials

Overall, Essic C + had the lowest Young’s modulus of elasticity and yield stress when compared to the other groups with means of 1007.6 MPa and 16 MPa, respectively. The Vivera group had the highest stiffness (2058 MPa and yield stress of 26 MPa), followed by Duran 1.5 (1713 MPa and 22 MPa). Zendura and Duran 1 mm had very similar outcomes with 1342 MPa for both groups and 18 to 16 MPa, respectively. The variation between the Vivera and Duran 1.5 mm in comparison to Essix C + group was found to be statistically significant (*P* < 0.01; Table 6).

**Table 6:** Linear regression analysis comparing Young’s modulus of elasticity between the five groups (with Essix C + as a comparator)

Young’s modulus	Coefficient	*P* value	95% confidence interval	
Essix C + (base comparison)	Reference			
Duran 1	334.6	0.09	− 64.20	733.34
Duran 1.5	705.8	< 0.01	307.04	1104.58
Vivera	1044.6	< 0.01	645.84	1443.38
Zendura	333.9	0.09	− 64.88	732.66

## Discussion

The VFR brands were selected to represent the three most popular chemical compositions (PP, PETG and PU), while also including PU variants given their novelty and the relative lack of associated research. A conventional design was tested in this study which did not include the palatal coverage in order to offer the most representative retainer design. The findings expose significant differences associated with VFR materials in terms of key physical properties. This knowledge can be utilized to tailor retention regimes where higher stiffness might be required; for example, to resist the tendency to maxillary arch constriction following active expansion during treatment. Similarly, designs less susceptible to wear could be considered in patients with parafunctional habits.

Previous studies used different methods and customized jigs to produce surface wear on thermoplastic materials. Raja et al*.* [11] investigated the wear resistance of PP and PETG retainers after thermoforming onto a template block and used metal rods with steatite balls attached to a wear machine with a load of 460 g for 1000 cycles. Gardner et al*.* [12] tested PP and PETG in a thermoformed state using a stone block (76 × 50 × 38 mm) followed by use of a two-body wear machine with steatite balls under a load of 25 kg for 1000 cycles of wear. Bratu et al. [13] used a custom jig, and upper and lower stone study models with retainers in place fixated on a metal plate with screws under a load of 61.2 kg for 10,000 cycles. The steatite ball possesses a hardness similar to tooth enamel (Mohs scale, 7.5) and is therefore more likely to induce a representative amount of wear on the thermoplastic material better simulating the intra-oral environment and related cycling. Furthermore, the use of block models for fabrication means that the thickness of the samples is uniform, while retainers tend to vary in thickness intra-orally; hence, breakages and perforation are seen in specific areas in the retainers with long-term wear [9, 14]. As such, a bespoke attachment with 10 mm steatite balls attached to the wear machine was used in the present study.

The samples used were thermoformed onto a 3D printed model based on our typodont model and then flattened to maintain the thickness variation of the materials in the molar-premolar region with the same sample dimensions.

In the hydrolysis test, previous studies have only tested thermoformed samples in distilled water over 2 weeks [15–17]. However, the water uptake between thermoformed and worn retainer samples had not previously been assessed. Therefore, in this study, a comparison was performed between thermoformed (control) and worn (experimental) groups in two media (de-ionized water and artificial saliva) with more prolonged follow-up incorporating five different time points up to 6 months.

Wear is considered the removal of material from a solid surface when undergoing mechanical interaction; however, clinical wear is a more complex process being influenced by normal function, parafunction and the effects of intra-oral cycling including temperature and pH change [18]. No material was resistant to wear with both Duran groups having less wear in comparison to Essix C + with a mean of 367 μm for the 1-mm group. These findings mirror those of Raja et al. [11] who found Duran to be 3.7 times more resistant to wear when compared to Essix C + . Moreover, Bratu et al*.* [13] found Duran to have 549-μm median wear in the lower arch. More significant wear levels in the latter study may relate to the increase in the load used (61.2 kg) and a higher number of cycles (10,000). PETG thermoplastic material was also proven to be more durable in terms of wear loss than PP [12], which agrees with our findings. This suggests that this retainer type may have lower levels of longevity and may be best avoided in those with known parafunctional activity.

Zendura had the highest amount of wear in the present study (652 μm). This was somewhat surprising as Vivera, which is a polyurethane material, similar to Zendura, had very minimal wear ~ (324 μm) comparatively. However, regardless of the amount of wear of these materials, visual inspection did not reveal obvious perforation of the tested samples. To date, there appear to be no studies that have compared the wear resistance of polyurethane Essix-type retainer materials. The present findings highlight the need to examine the material properties of these relatively novel retainer variants in more detail and in the clinical environment.

Young’s modulus and yield stress were lowest in Essix C + (PP) followed by Zendura (PU) and Duran 1 (PETG), then Duran 1.5 (PETG), and finally Vivera (PU) which had the highest values for both properties. These findings reflect those reported in the literature [16, 19] with PU having the highest stiffness followed by PETG and then PP with the least stiffness. Furthermore, Tamburrino et al*.* [20] compared Zendura and Duran in thermoformed states and following 7 days of immersion in artificial saliva finding only a 37-MPa difference between the two groups. Although the observed results are higher than those recorded in the present study, this may relate to the differences in sample dimension and the thickness of the blank sheets. The results for tensile strength with Duran were higher than those observed by Ahn et al*.* [21], which may relate to their use of a thinner dimension (0.8 mm vs. 1.5 mm and 1 mm in our study).

In terms of the hydrolytic absorption and degradation, uptake differed from one material to the next as Essix-type retainer materials have varying levels of crystallinity, which ultimately may have an effect on their molecular structure. Essix C + had the highest absorption value in the control group (thermoformed) with 15% in artificial saliva after 6 months, while Zendura had the highest absorption value of 15 wt% in the experimental group, followed closely by Essix C + after 6 months of water absorption. These outcomes differ from Ryokawa et al. who monitored the water absorption of thermoformed samples at different timelines up to 2 weeks of immersion. PU samples absorbed the highest amount at 1.5wt% followed by PETG with 0.85 wt% and PP at 0.12wt%. Similarly, Inoue et al*.* observed that PP had the least amount of absorption [15, 16]. Although propylene is a hydrophobic polymer, variations in the immersion time (2 weeks vs. 6 months) and medium (water vs. artificial saliva) as well as differences in the drying processes (drying with cloth vs. filter paper) may have a bearing on the observed findings.

Based on the FTIR spectra, the degree of absorption seen with Essix C + suggests some alteration to the molecular compounds compared to the initial scan. Polypropylene is a linear hydrocarbon polymer with a chemical structure (CH_2_ = CHCH_3_). In the FTIR, an additional absorption broad O–H group stretch at 3300 cm^−1^ and absorption at 1611 cm^−1^ assigned to the stretching vibration of the carbonyl (C = O) group were noted, post-testing and post-drying. These were not detected in other materials including Zendura (PU). Our findings were similar to Ahn et al. [21] who confirmed no changes were seen in the surface structure of the materials following 6 months of wear in vivo for PETG thermoformed retainers. However, they did detect new elements including silicone (Si), phosphorus (P) and calcium (Ca) after EDX spectroscopy which we have not included in this study. The stability of the chemical structure in both PU groups in this study is in keeping with Bradley et al. [22] who investigated the chemical and mechanical change of PU aligners after 44 ± 15 days of wear. However, further studies are required to investigate the different types of polymers and their degradation rates with long-term surface changes pertaining to removable orthodontic retention.

## Limitations

The in *vitro* design used in this study cannot fully account for intra-oral variables such as variation in saliva composition. Furthermore, significant variability in terms of intra-oral conditions is known to occur clinically with Gibbs et al*.* [23] reporting that the average occlusal force of posterior teeth upon closure can be elevated in those with parafunctional activity. The maximal force utilized did not exceed 4.6N (470 g) being similar to force levels used in previous research [10]. Higher loads (245 N/25 kg and 600 N/61.2 kg) with similar numbers of wear cycles (2000 and 10,000) were used in other studies with no signs of perforation or occurrence of tear points [11, 12]. There is a need for further studies investigating the effects of localized excessive surface wear on Essix-type retainers. It would be useful to include variable force levels and patterns within these experiments.

## Conclusion

- Essix C + (PP) was found to be the least stiff material with Vivera (PU) having the highest level of stiffness.
- All materials were susceptible to water absorption; however, the chemical structures were stable in all groups with the exception of Essix C + (PP).
- Duran 1.5 mm (PETG) performed similarly to Vivera (PU) in terms of stiffness and wear properties.
- While Zendura and Vivera have similar chemical structure (PU), they performed differently in terms of wear resistance and water absorption.
- Further clinical research is required to validate the present findings and to understand the effectiveness and longevity of a range of Essix-type retainer materials.
